# Professional Success

**Published:** 1861-05

**Authors:** A. S. Talbert

**Affiliations:** Lexington, Kentucky


### PROFESSIONAL SUCCESS.

BY A. S. TALBERT, D. D. S., LEXINGTON, KENTUCKY.

Read before the Kentucky State Dental Association.

*Gentlemen of the Kentucky State Dental Association* :—In the fulfillment of a duty imposed upon me at our last meeting, I present you a few thoughts on some of the elements of professional success. To insure success in any undertaking, the means must be adequate to the end to be attained. The adaptation of the means to the end, so as to accomplish the desired object may, then, be defined *a success*.

It is the common lot of man to labor, either mentally or physically for the supply of his natural wants. It is an essential element of his nature. It is a fundamental law of his being. It is the inevitable decree of God: "In the sweat of thy face thou shalt eat bread."

But his labors are not confined to the same narrow sphere. To him alone is given the capability of eternal progress. To him alone is given the power to profit by the past, or to anticipate the future. He alone is endowed with inventive genius. He alone subjects the elements to his will, and controls them to do his bidding.

*Our* eagle builds his eyry no higher nor perches himself more proudly on his mountain throne than did his fathers forty centuries before him. His ærial couch is made after the exact pattern as that of his illustrious ancestor's! *Our* lion's roar is not more terrible, nor the hind's foot more nimble, than when Daniel brooked the fierceness of the one, or when God inquired of Job if he knew the ways of the other. *Our* birds do now make the same toilette which they wore at their matineés in the days of St. Valentine. The sweet, and ever varied notes of the mocking-bird have lost none of their melody, nor has the owl added to his wisdom by the teaching of ages!

Man alone reasons—man alone thinks. He who thinks with the most system, suiting the *action* to the thought, will, *cæteris paribus*, soonest attain to the object desired.

There is no excellence without labor, neither is there labor without reward. Even the smallest force brought to bear on any object an indefinite length of time will produce a definite result. There is perhaps no avocation to which these truths are more applicable than to the profession of Dentistry. Called into existence by the ills to which the teeth are subject, it undertakes to alleviate suffering—to arrest the progress of disease, and to supply a substitute for the lost member. To accomplish these three great objects, a versatility of talent and a firmness of purpose, coupled with correct judgment, are required: to which add a good moral character, and a thorough Dental education, and our student is qualified to assume the responsibilities of his profession.

It is now necessary to provide a suitable office in which to receive his patients, and to perform his operations. Three or four rooms will suffice for this purpose. These rooms should be well lighted and ventilated;—easy of access and neatly furnished;—the operating room being supplied with a washstand and its necessary appurtenances,—an operating chair and accompaniments—an instrument case and suitable instruments, in the skillful use of which it must be admitted lie the foundations of success. Ours is a tangible profession. The larger number of our operations exhibit for themselves the skill or the blunders of the operator. We can not hide our mistakes behind technicalities. We can not throw around them a woof to veil their deformities! We can not *bury our dead!* We have no magic wand that, with soothing enchantment, will conjure nature to restore a lost plug, or cement a broken tooth. We deal with solid realities. Nor can we shrink from the responsibility of any operation; for we can not operate by proxy. We live literally "from hand to mouth!" But while it is essentially true that the foundation of success lies in the excellence of the operation, that alone will not always insure success. We have to deal with delicate tissues, as well as "solid realities." Force should always be applied in a direct ratio to the strength of the resisting object. While a tooth must be plugged in accordance with this law, it must at the same time be done with the strictest possible regard to the comfort of the patient.

Let the lips and mouth be handled with the gentlest touch of *clean fingers*, and the operation be performed with clean, well burnished instruments. There is no part of our system so delicately organized as are our mouths. None, for which we have so scrupulous a regard to cleanliness. We may touch with impunity a loathsome object to any other part of our bodies, that would disgust and sicken us if applied to our mouths! That we meet with "*bad mouths*," no one will deny; but the continued neglect of cleanliness on the part of our patients does not offer any just pretext for a like neglect on our part.

I will give you an example to show you how little things may become important elements of success. A servant girl once called at my office to have me extract a tooth. I seated her in the chair, and, having washed my hands as I always do before going to my patient, I performed the operation. A few months after, her mistress called on me, and, paying a handsome fee for professional services, she became ever after while she lived, my steadfast friend. She told me she came at the instance of her servant, who said she "never before saw a *white man wash his hands to wait on a nigger!*" Thus a simple act of kindness (for it was nothing more—my hands being as clean as her mouth,) shown even to a slave was a hundred fold rewarded, adding more to the *success* of the operation than any superior skill could possibly have done.

As material substances depend on an aggregation of many homogeneous particles, so success depends on many things, apparently little in themselves, yet each of momentous importance to the formation of a perfect whole. Little courtesies often give to men of inferior minds an influence and a business that are denied to their superiors. They are especially fitting to our profession; many of our patients do not come to us till they are driven by the severest necessity. Their system being unnerved by pains and loss of sleep, they are unable to demean themselves with their wonted composure. Kindness will give them confidence—confidence in you, confidence in themselves. It will inspire in them respect for you, and they will bear their pains with becoming resignation. Having properly completed your operation, it is a success,—your association with your patient is a success. You have fulfilled a professional duty,—you have gained a friend. Nor is kindness alone an element of success; it must be accompanied with dignity, candor and honesty. No tricks of legerdemain are to be practiced, nor are very intimate social relations required; from a few minutes to as many hours will suffice for the exercise of these relations. Our offices are not a fit place for social intercourse, they are for *business*. While in them we are to treat all with the same respectful consideration during the time required for professional services. But it is not our duty to offer inducements to any to remain longer. Do not therefore urge them to stay,—you do not know at what moment a patient may call whom you would prefer to meet alone: you do not want *either* to hear the *other's* complaints or commendations. Have you not had Mr. A——, whom you have not seen for *three years*, come in just as you were making an engagement with Miss B——, and say in her presence, "Doctor, one of the plugs you put in for me *last year*, came out a few days ago, when in fact it had never been filled by any one; or, perchance had been filled many years before by some other Dentist!

Miss B—— in the meantime has left with an unfavorable impression, strengthened it may be by some casual remark of some admirer of your neighbor dentist, and *possibly* you see nothing more of her!—for our patients do not *always* fulfill their engagements. But this fact does not afford any pretext for the neglect of *our* engagements.

Punctuality is a jewel in the crown of our success. We must not trifle with our engagements, nor allow either pleasure or business to break them with impunity. We are the servants of the sovereign people, *they* fully recognize the relationship. *They* may break their engagements for some trivial pretence, once, twice, or three times, but *we* must not break ours on the fourth time; no, no, *we* belong to them! Our only redress is to come and go, or rather always *stay* in our offices, to do their bidding; but we may see to it that it is done *at their expense!* This reminds us of another important element of success, to-wit: properly regulated professional fees,—they should be just and equitable in view of the services rendered, the ability of the patient, and our general professional responsibilities. We can not always estimate our services by the amount of time, or the quantity of material used.

It is often necessary to fill a tooth which we know can not long be of service, but to do which it may require more material and labor than to fill another which will be good during the life of our patient. It would be unjust to charge more for the first than the second, because it is intrinsically of less value, and yet we may charge a fair compensation for any operation competent to be performed, and that is as well done as may be under the circumstances. So too, in many of our minor operations,—little in themselves, they seem to us hardly worth charging for. But it must be remembered, that what seems to us little, and very easily accomplished, is rendered so only by familiarity; for when a thing is thoroughly learned, it seems very easy to know it! and we often wonder we had been so stupid as not to have learned it sooner! If then our benevolence incline us to charge nothing for what appears to us a trifling operation, it is well to remember our superior knowledge and skill have been attained by study and experience, for which we are justly entitled to fair compensation. But it is our province to use a prudent discretion in making our charges; all are not equally able to pay the same prices. The poor, as well as the rich, require our services,—the *little* they are able to pay is even more to them, than the *much* paid by the wealthier classes is to them ; hence they are entitled to equally good and permanent operations ; indeed, to better if possible, as they are less able to pay for their repetition. We should have no "cheap Dentistry" at "cheap prices," but good Dentistry at low prices, in all cases where our patients are not able to pay *remunerative* prices.

Much of success depends upon the *manner* of our operating ; it is not to be done hurriedly, nor are we to wear out our patients by too long sittings. Again, there are those for whom it is well to operate *when you have the opportunity!* A correct discrimination as to the *time* is equally as important as the *manner* of operating. "There is a time for every thing under the sun." Have your instruments in proper order before your patient sits in the chair, that the sharpening may not grate harshly on his ears, or the cleansing be associated with their previous use. Listen to your patient's wishes ; have respect to them, but consult your own interest and that of your profession first, both of which being combined are paramount to his. If his teeth are to be filled, he will say to you, "fix the worst ones first!" Are you to do it? By *no means!* A little *placebo* may be necessary ; a little camphor and cotton ; make the application, and by the time you have filled the smaller cavities, your camphor will have done wonders! But how? Not so much in its having "toughened the inflamed dentine," as in your having obtained the confidence of your patient and in his better control of his nerves, by which he will better bear the operation, and so afford you an opportunity to complete it in the best possible manner ; whereas, if you had commenced with the "worst ones," your patient's courage might have failed him by the time *they* were completed. Do not then begin with "*the worst*," for by so doing you take *all the risks*,—among which may be mentioned the larger quantity of material required—the more time consumed, and the greater liability of the teeth to furnish cause for complaint by their aching, or sensibility to changes in the temperature of the food and drinks.

Let the same rule be observed in the extraction of a number of teeth, commencing with the one most easily drawn, and you will thereby inspire confidence and give courage.

Thus, gentlemen, I have rapidly glanced at some of the elements of success.

I thank you for your kind attention, regretting I have not been able better to entertain you.

